# Caecal volvulus in an adult with an incomplete common mesentery: A case report^☆^

**DOI:** 10.1016/j.ijscr.2023.108353

**Published:** 2023-05-24

**Authors:** Dorra Bel Haj Yahia, Souhaib Atri, Amine Sebei, Youssef Chaker, Houcine Maghrebi, Montasser Jameleddine Kacem

**Affiliations:** The Faculty of Medicine of Tunis, University of Tunis El Manar, Tunis, Tunisia; Surgery Department A, RABTA Hospital, Rue Jbel Lakhdar, La Rabta Jebbari 1007, Tunis, Tunisia

**Keywords:** Common mesentery, Intestinal malrotation, Caecal volvulus, Case report

## Abstract

**Introduction and importance:**

A common mesentery is defined by the persistence of an embryonic anatomical arrangement secondary to an anomaly of rotation of the primary umbilical loop. Caecal volvulus is a rare cause of intestinal obstruction, which account for 1 to 1.5 % of all intestinal obstructions. A combination of both, intestinal mal rotation and caecal volvulus is rare.

**Case presentation:**

We report this rare entity in a 50 year old male with no history of abdominal surgery who was admitted for an acute intestinal obstruction. Clinical examination found a non-complicated right inguinal hernia. Radiological assessment showed signs of an incomplete common mesentery and an important small bowl distention with a transitional zone near the profound inguinal ring. Emergency surgery was performed. Surgical exploration didn't find signs of strangulation in the inguinal hernia which motivated midline laparotomy. We discovered a caecal volvulus with an incomplete common mesentery and ischemic lesions in the caecum. Ileocaecal resection was performed with ileocolostomy.

**Discussion:**

Common mesentery can be complete or incomplete. It is often well tolerated in adulthood. This intestinal malrotation can sometimes cause serious complications such as volvulus. Their association is rare. Radiology can be very helpful in leading to the diagnosis, but the diagnostic process should not delay surgical intervention which is the basis of the treatment.

**Conclusion:**

Caecal volvulus is a serious complication of intestinal malrotation. This association is rare in adulthood and symptoms are not specific. Emergency surgery is necessary.

## Highlights

• The association of caecal volvulus and intestinal malrotation is rare.
• CT scan is mandatory for preoperative diagnosis of caecal volvulus.
• Emergency Surgery is necessary.
• The treatment is based on three elements: reducing torsion, treating possible complications and preventing recurrences.

## 1 Introduction

A common mesentery is a result of an abnormal rotation of the primary umbilical loop. It can be complete or incomplete at a 180°. It is characterized by a persistence of an embryonic anatomical arrangement with a common *meso* to the entire intestine and a short root of the mesentery [1].

It is mostly asymptomatic and there for it's estimated prevalence in adulthood is around 0.2 % to 0.5 % [2]. It can sometimes cause serious complications such as volvulus.

Caecal volvulus is a rare cause of intestinal obstruction, which account for 1 to 1.5 % of all intestinal obstructions. It is defined by an axial torsion of the caecum, ascending colon, and terminal ileum around the mesenteric vascular pedicles [3]. A combination of both intestinal mal rotation and caecal volvulus is rare.

While radiology can be very contributive to the diagnostic process, it can sometimes fail to deliver the final diagnosis. Emergency surgery should not be delayed as surgery is the main and most recognized treatment.

We report a rare case of a scarce association of a caecal volvulus and an incomplete common mesentery in a 50 year old male.

This case report has been reported in line with the SCARE Criteria [4].

## 2 Case description

A 50 year old male with chronic kidney disease in the terminal stage receiving hemodialysis and no history of abdominal surgery or bowel disorder was admitted in our surgery department for acute abdominal pain with a rapidly progressive intensity which evolved for 24 h, with nausea, vomiting and abdominal distension. At the initial physical examination, the patient had stable vitals and had no fever, but he was agitated with a poor general condition. The Abdominal examination found a distended tympanic abdomen, with a generalized tenderness, and a large, irreductible but painless right inguinal hernia, which was against the diagnosis of an intestinal obstruction due to a strangled hernia. Rectal examination found an empty rectal bulb. Biology assessment showed a normal white blood cell count but an elevated level of C-Reactive Protein (170 mg/L).

**Fig. 1:**
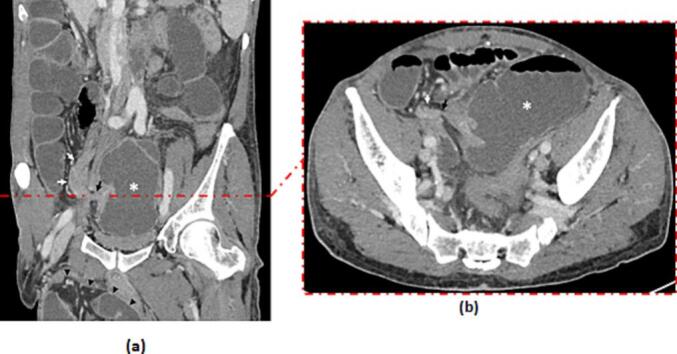
Coronal (A) and axial (B) enhanced CT images showcase a dilated caecum (white asterisk) located in the left iliac fossa and pelvis converging and tapering into a transitional point located in the right iliac fossa (black arrow) without dilation of the right colon (white arrows) nor the rest of the colonic segments. Note the right scrotal hernia (black arrow-heads) and the distension of the small bowl.

**Fig. 2:**
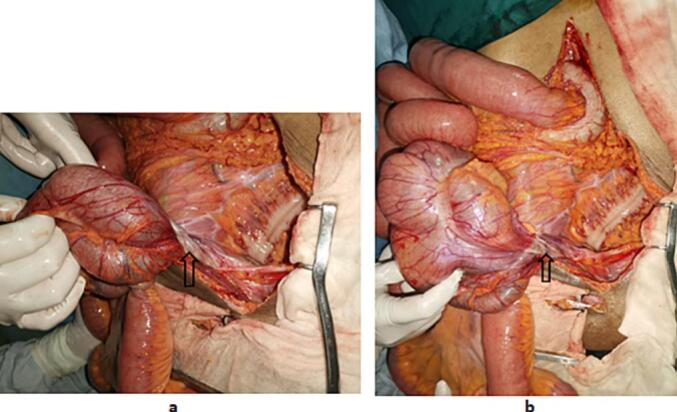
peroperative view of the caecal volvulus (**a** and **b**). Black arrow: twist zone.

**Fig. 3:**
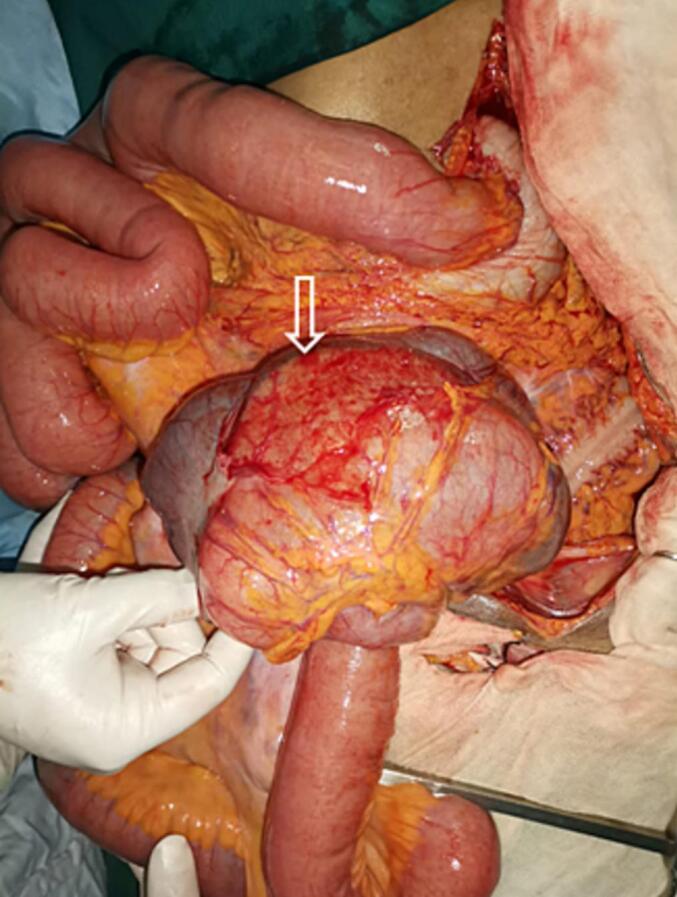
Ischemic lesions of the caecum (white arrow).

After conditioning and reanimation, a radiological assessment was done. Abdominal x-ray showed air-fluid levels. Abdominal CT scan revealed an abnormal position of the duodenojejunal (D-J) flexure which was located to the right of the midline, the third portion of the duodenum was not included in the arterial clamp. The caecum was in the midline in a transverse position, in contact with the anterior abdominal wall with an important distention of small bowel and a transitional zone next to the deep inguinal ring (Fig. 1). We decided to perform an emergency laparotomy by a senior surgeon. We proceeded by a right inguinal incision. Exploration showed distended small bowel with no restriction signs at the deep inguinal ring. We then decided to proceed to a midline laparotomy. We found caecal volvulus with a coecum located in the midline and an important distention of small bowel which were located at the right side of the abdomen (Fig. 2). There was also an abnormal position of the duodenojejunal (D-J) flexure which is located to the right of the midline (normally to the left). The final diagnosis was therefore a caecal volvulus associated with an incomplete common mesentery. Ischemic lesions were noticed in the caecum (Fig.3). W performed an ileo-caecal resection with an ileostomy and a colostomy in the right iliac fossa, we preferred not to do an immediate anastomosis, as there was an important edema of the bowel wall. A suture repair of the right inguinal hernia was also performed. Post-operative course was marked by hyperkaliemia that needed hemodialysis. The patient was discharged after 20 days. Pathology showed signs of chronic ileocolic ischemia.

## 3 Discussion

Common mesentery results from an abnormality in the rotation of the digestive tract and this insufficient rotation is most often associated with a lack of attachment [1]. The rotation stop of the yolk loop at 90° makes the complete common mesentery with a colon located on the left, mesentery and small bowel on the right, but the most frequent form of intestinal rotation anomalies, is the incomplete common mesentery. That corresponds to a stop of the yolk loop at 180° and the cecum comes to join in the sub-hepatic region, which explains the higher risk of volvulus [5]. Caecal volvulus is an uncommon cause of intestinal obstruction. It affects the ascending colon and the terminal part of the ileum which are twisted around the mesenteric pedicle. It causes strangulation, hence, an occlusion of the two ends of the volvulated segment compromising its blood supply, which causes an obstruction in a closed loop [6,7].

As the persistence of the primary common mesentery in adulthood is often well tolerated and it can rarely be responsible for volvulus, few studies on this acute complication in adults have been reported in the literature, which is why our case is interesting. Clinical manifestations are not specific. The cecum volvulus produces a clinical table of acute intestinal obstruction by strangulation. Abdominal x-ray may show a large hydroaeric level reflecting the distension of the cecum, that could be median, lateralized to the right or to the left [1], but that is an inconstant sign. CT-scan can show a pathognomonic whirl sign demonstrating the caecal volvulus with signs of an incomplete common mesentery such as the absence of a retromesenteric D3 segment of the duodenum, abnormal superior mesenteric artery (smaller and more circular) – Superior mesenteric vein relationship and large bowel predominantly on the left and small bowel predominantly on the right [8]. In our patient, although imaging showed signs of incomplete common mesentery, yet it failed to deliver the final diagnosis of a caecal volvulus that is probably due to the presence of an inguinal hernia with a transitional zone next to the deep inguinal ring.

Unlike sigmoid volvulus, endoscopic treatment is not of much interest, with a failure rate exceeding 75 % of cases, but it could be interesting in the case of a contraindication to surgery or in risky areas [9]. The treatment is based on three elements: reducing torsion, treating possible complications and preventing recurrences. It is essentially surgical using the conservative technique LADD procedure (detorsion, cecopexy by fixing the cecum to the posterior peritoneum). In our case, as there were ischemic lesions in the caecum, conservative surgery was not possible. We performed an ileocaecal resection and decided to delay the ileocolic anastomosis.

## 4 Conclusion

Ceacal volvulus is a serious complication of intestinal malrotation. This association is rare in adulthood and symptoms are not specific. Ct scan is very important to make the preoperative diagnosis. However, it can sometimes mislead surgeons. Emergency surgery should not be delayed, and the surgical technique depends on peroperative findings.

## Patient's consent

Written informed consent was obtained from the patient for publication of this case report and accompanying images. A copy of the written consent is available for review by the Editor-in-Chief of this journal on request.

## Ethical approval

The work has been approved by the ethical committee related to the institution (Rabta hospital) CHL n°056 on May 8th 2023.

## Funding

No funding was granted.

## Author contribution

Dorra bel haj yahia: Conceptualization Writing – original draft.

Souhaib Atri Writing – original draft.

Amine Sebei data curation.

Youssef Chaker conceptualization.

Houcine Maghrebi supervision.

Jameleddine montasser kacem supervision-validation.

## Guarantor

Dr. Dorra Bel Haj Yahia.

## Research registration number

None.

## CRediT authorship contribution statement

**Dorra Bel Haj Yahia:** Conceptualization, Writing – original draft. **Souhaib Atri:** Writing – original draft. **Amine Sebei:** Data curation. **Youssef Chaker:** Conceptualization. **Houcine Maghrebi:** Supervision. **Montasser Jameleddine Kacem:** Supervision, Validation.

## Declaration of competing interest

The authors have no conflicts of interest to declare.

## Data Availability

None.
